# Deciphering the Costs of Reproduction in Mango – Vegetative Growth Matters

**DOI:** 10.3389/fpls.2016.01531

**Published:** 2016-10-21

**Authors:** Mathilde Capelli, Pierre-Éric Lauri, Frédéric Normand

**Affiliations:** ^1^CIRAD, UPR HortSys, Saint-PierreReunionIsland, France; ^2^Inra, UMR SystemMontpellier, France

**Keywords:** allocation of reproduction, flowering, fruiting, growth unit, irregular bearing, *Mangifera indica*, tree architecture

## Abstract

Irregular fruit production across successive years is a major issue that limits the profitability of most temperate and tropical fruit crops. It is particularly affected by the reciprocal relationships between vegetative and reproductive growth. The concept of the costs of reproduction is defined in terms of losses in the potential future reproductive success caused by current investment in reproduction. This concept, developed in ecology and evolutionary biology, could provide a methodological framework to analyze irregular bearing in fruit crops, especially in relation to the spatial scale at which studies are done. The objective of this study was to investigate the direct effects of reproduction during a growing cycle on reproduction during the following growing cycle and the indirect effects through vegetative growth between these two reproductive events, for four mango cultivars and during two growing cycles. Two spatial scales were considered: the growth unit (GU) and the scaffold branch. Costs of reproduction were detected between two successive reproductive events and between reproduction and vegetative growth. These costs were scale-dependent, generally detected at the GU scale and infrequently at the scaffold branch scale, suggesting partial branch autonomy with respect to processes underlying the effects of reproduction on vegetative growth. In contrast, the relationships between vegetative growth and reproduction were positive at the GU scale and at the scaffold branch scale in most cases, suggesting branch autonomy for the processes, mainly local, underlying flowering and fruiting. The negative effect of reproduction on vegetative growth prevailed over the positive effect of vegetative growth on the subsequent reproduction. The costs of reproduction were also cultivar-dependent. Those revealed at the GU scale were related to the bearing behavior of each cultivar. Our results put forward the crucial role of vegetative growth occurring between two reproductive events. They are discussed in the context of irregular bearing considering both the spatial scale and the various bearing habits of the mango cultivars, in order to formulate new hypotheses about this issue.

## Introduction

Fruit production in most tropical and temperate perennial fruit crops is irregular across successive years. Irregular bearing is characterized by years of high fruit production (‘on’ years) and by years of low fruit production (‘off’ years) (Monselise and Goldschmidt, 1982). The particular pattern of regular alternation of ‘on’ and ‘off’ years is referred to as alternate bearing. Since some fruit traits are related to yield, irregular bearing affects fruit quality across years and has economic consequences for the fruit industry. Research has been carried out to identify factors that trigger and factors that maintain irregular bearing (Monselise and Goldschmidt, 1982; Bangerth, 2009), and to understand the physiological mechanisms involved in this phenomenon (Wilkie et al., 2008; Muñoz-Fambuena et al., 2011; Samach and Smith, 2013; Smith and Samach, 2013). Several studies suggest that irregular bearing is related to the balance between various types of resources (e.g., carbon/nitrogen ratio) and to hormonal factors (auxin, cytokinins, gibberellins) at the scales of the whole tree and of the shoot (Chan and Cain, 1967; Marino and Greene, 1981; Goldschmidt and Golomb, 1982; Rosecrance et al., 1998). Within a given species, cultivars differ in their bearing behavior (Monselise and Goldschmidt, 1982; Knight et al., 2009), and recent studies evidenced the genetic control of irregular bearing in apple (Guitton et al., 2012; Durand et al., 2013).

Studies on nutritional and hormonal mechanisms focus mainly on the effects of fruit production one year on flowering and/or fruiting the following year, and do not take the vegetative growth that occurs between two reproductive periods into account. Nevertheless, negative relationships has been shown between reproduction and vegetative growth in olive (Connor and Fereres, 2005; Castillo-Llanque and Rapoport, 2011), apricot (Costes et al., 2000), apple (Lauri and Térouanne, 1999), avocado (Lovatt, 2010), and peach (Berman and DeJong, 2003). On the other hand, characteristics of vegetative growth can affect reproduction in various fruit species, including apple (Lauri and Trottier, 2004) and mango (Normand et al., 2009). These results suggest that vegetative growth could be involved in irregular bearing. Dambreville et al. (2013) identified architectural factors (e.g., apical vs. lateral position of the shoot) and temporal factors (e.g., date of burst of the shoot) in the mango tree that are involved in the reciprocal interactions between vegetative growth and reproduction at the shoot scale. They showed significant interplay between structural and temporal components of architectural development with significant positive or negative relationships between successive shoots within and between growing cycles. For example, flowering or fruiting delays vegetative growth during the following cycle, and late vegetative growth decreases the probability of flowering. These relationships then appear as a key point to describe and decipher irregular bearing in fruit trees.

The concept of the costs of reproduction developed in evolutionary biology and ecology is defined in terms of losses in the potential future reproductive success caused by the current investment in reproduction (Jönsson, 2000). Two types of costs of reproduction can be distinguished: the direct costs, corresponding to the direct investment in flowering and fruit growth during the current reproductive season, and the indirect or delayed costs of reproduction, corresponding to the effects of reproduction on the subsequent vegetative growth that, in turn, can affect reproduction (Newell, 1991; Obeso, 2002). The costs of reproduction can be evaluated from a nutritional point of view, for example by determining the carbon and nitrogen costs associated with vegetative and reproductive growth (Daniels et al., 2013), as well as from a demographic point of view, e.g., the higher the number of flowering buds is, the lower the number of vegetative buds for vegetative growth. The hypothesis behind the costs of reproduction is that compromises are necessary to allocate plant resources to three main vital functions, namely growth, reproduction and defense, in order to maximize the reproductive success during the entire life span of the plant and not just during one growing cycle (Obeso, 2002).

In fruit crops, the negative effects of reproduction on vegetative growth (see references above) and subsequent flowering (Chan and Cain, 1967; Marino and Greene, 1981; Lovatt, 2010) appear as delayed costs of reproduction. But only rare studies use explicitly the concept of the costs of reproduction and its associated methodologies (e.g., Stevenson and Shackel (1998) for pistachio, *Pistacia vera*). Yet, the costs of reproduction are expected to be higher in fruit crops and easier to detect because of the selection of genotypes with high yield, i.e., with higher allocation to reproduction (Obeso, 2002). Moreover, although the costs of reproduction are often studied at the whole plant scale, they may or may not be detected at lower scales within the tree (shoot, branch), in particular, in relation to branch autonomy (Sprugel et al., 1991; Obeso, 1997, 2004).

Mango (*Mangifera indica* L.) is a monoecious evergreen species. It rates fifth in terms of worldwide fruit production (Gerbaud, 2015), and is one of the major fruit crops in tropical areas (Purseglove, 1972). Mango fruit is very important for people living in tropical countries at both the nutritional and economic levels (Mukherjee and Litz, 2009; FAO, 2011). The mango tree is an irregular bearer with a cultivar-dependent pattern of irregular bearing: some cultivars are relatively regular in terms of fruit production across years, whereas others have an irregular or alternate fruit production (Chacko, 1986; Dambreville et al., 2014).

The objective of this study was to investigate the vegetative and fruiting behavior of four mango cultivars during two growing cycles in order to evidence the costs of reproduction in mango and to determine how they could explain irregular bearing. We considered a demographic, and not a nutritional, approach of the costs of reproduction in order to propose different hypotheses about the nature of the mechanisms (trophic, hormonal, …) underlying the results. Our two hypotheses were that a higher reproductive effort during one growing cycle led to lower vegetative growth during the following cycle, and that this lower vegetative growth led to reduced reproduction. Our specific objectives were to answer the following questions: (i) What are the effects of the investment in reproduction during a growing cycle on reproduction during the following growing cycle? (ii) Are these effects mediated by the effects of reproduction on vegetative growth? (iii) At which scale within the tree [growth unit (GU), scaffold branch] do these effects occur? and (iv) Are these effects cultivar-dependent?

## Materials and Methods

### Plant Material and Experimental Setup

The experimental orchard was located at the CIRAD (French Agricultural Research Center for International Development) research station in Saint-Pierre, Reunion Island (21°31′ S, 55°51′ E, 280 m a.s.l.). It was composed of eight mango cultivars with 14 trees per cultivar, all grafted onto the polyembryonic rootstock ‘Maison Rouge’ and planted in May 2001. We chose to study four of these cultivars with contrasted patterns of irregular bearing in the orchard (unpublished data): José, a local cultivar from Reunion Island characterized by a strong irregular bearing; Cogshall, a Floridian cultivar that is extensively grown in Reunion Island and which is characterized by a weak irregular bearing; Kensington Pride, the main cultivar grown in Australia with a quite regular productivity in Reunion Island; and Irwin, a Floridian cultivar, the most regular bearer among the four cultivars studied. We studied three trees per cultivar. They were not pruned before or during the experiment to avoid any effect of manipulation on their vegetative development and reproduction.

**FIGURE 1:**
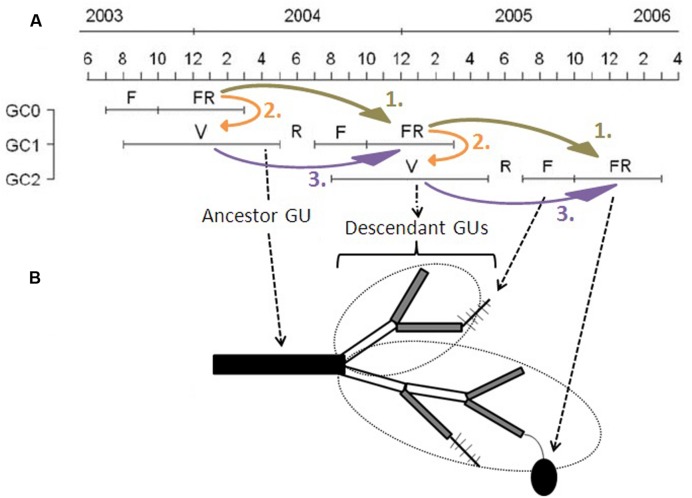
**Scheme of the partial overlapping of three consecutive growing cycles in mango. (A)** Temporal succession of the three growing cycles (GC0, GC1, and GC2) of mango trees studied for this work from June 2003 to April 2006. Each GC is composed of a succession of a vegetative growth period (V), a rest period (R), flowering (F), and fruiting (FR). **(B)** Succession of growth units (GUs) during two growing cycles. The ancestor GU related to GC1 is represented by a black rectangle, and the descendant GUs of GC2 by gray (terminal GUs) or white (non-terminal GUs) rectangles. Leaves are not represented. Fishbone-like symbols are inflorescences, and the black ellipse is a fruit. In this scheme, there is one ancestor GU that produced eight descendant GUs, making up two axes, circled by dotted ellipses, one with three GUs and one with five GUs. Among these descendant GUs, there are five terminal GUs. Two of them remained vegetative, the other three flowered and, among them, one set one fruit. Curved and colored arrows represent the three complementary steps of data analyses. Adapted from Dambreville et al. (2013).

The growing cycle (referred to as ‘cycle’ hereafter) of the mango tree lasts about 18 months in Reunion Island and is composed of four main phenological stages (Dambreville et al., 2013): vegetative growth (from August in year n-1 to April in year n), rest period (May to July in year n), flowering (August to October in year n), and fruiting (fruit growth and maturity, from December in year n to February in year n+1) (**Figure 1A**). Vegetative growth may begin from the second half of the flowering period to the end of fruit growth of the previous cycle and continue after the harvest during the hot and wet season. Consequently, cycles overlap in a single tree, with part of vegetative growth during the reproductive period of the previous cycle, from August to February (**Figure 1**). Vegetative growth is rhythmic and asynchronous within and between trees, and is related to the appearance of new GUs, defined as the portion of the axis developed during an uninterrupted period of growth (Hallé and Martin, 1968; Barthélémy and Caraglio, 2007), at different dates, usually called “flushes.” Floral induction of mango occurs just before the burst of inflorescences (Davenport, 2009), about 7–8 months after the previous fruit harvest. Mango flowering is made up of inflorescences that appear at the tip of terminal GUs (terminal flowering). Only some inflorescences set fruits.

Kinship terms are used to describe the succession between GUs. The last GU developed during a given cycle is referred to as the ancestor GU. New GUs produced during the following cycle from a single ancestor GU are referred to as its descendant GUs. All descendant GUs from a single bud of an ancestor GU during a cycle form an axis, possibly including branching. Among the descendant GUs, those in terminal position are able to flower and set fruit during the current cycle (**Figure 1B**). Descendant GUs in terminal position during a cycle are the ancestor GUs for the following cycle. The ancestor GUs are the focal points of this study because they bear the reproduction of the cycle and are the starting points for vegetative and then reproductive development during the following cycle.

### Data Collection

The experiment was carried out over two growing cycles, from August 2003 to February 2005 (cycle 1), and from August 2004 to February 2006 (cycle 2). Trees were first harvested at the beginning of the study, i.e., from December 2003 to February 2004 (cycle 0). The terminal GUs of each tree were identified in June 2003, before flowering of cycle 0. The scaffold branches to which they were connected were recorded. For each cycle, the fate [vegetative: V (did not flower); flowering: F (flowered but did not set fruit); fruiting: FR (flowered and set fruit)] of each terminal GU was recorded during the flowering and fruiting period. These terminal GUs were the ancestor GUs for the following cycle. The number of fruits and fruit mass were recorded per fruiting terminal GU during the three cycles 0, 1, and 2.

During the rest period of cycles 1 and 2, the basal diameter of all axes developed from ancestor GUs was recorded after the complete extension of the terminal descendant GUs. The number of terminal and non-terminal GUs was recorded for each axis. The leaf area of each axis was estimated from its basal diameter with allometric relationships (Normand and Lauri, 2012). The basal diameter of the trunk and of each scaffold branch of the trees was measured during flowering of cycles 0, 1 and 2, and converted into cross-sectional area assuming a circular section: BCSA (branch cross-sectional area) for scaffold branches and TCSA (trunk cross-sectional area) for trees. Leaf area, the number of terminal GUs and the number of fruits and fruit mass collected at the scale of the ancestor GU were then aggregated at the scale of the scaffold branch.

### Data Analysis

Data were analyzed for each cycle. Vegetative growth produced by an ancestor GU was quantified by two main variables corresponding to two complementary points of view: the number of terminal descendant GUs, hereafter referred to as ‘terminal GUs,’ produced during vegetative growth; and the leaf area of descendant GUs. The number of terminal GUs gives a demographic point of view that represents the part of the vegetative growth that is able to flower and possibly set fruit. It is therefore an architectural trait that represents the potential for subsequent reproduction. The leaf area of descendant GUs has an ecophysiological significance and gives a proxy of local carbohydrate availability linked to the capacity of the plant to capture light and, therefore, to photosynthesize. It is also allometrically related to the stem mass and volume of the descendant GUs (Normand et al., 2008) and, consequently, to the capacity of local storage of carbohydrates for subsequent reproduction. At the scaffold branch scale, vegetative growth was quantified by aggregated data for these two variables, normalized by the size of the scaffold branch, i.e., divided by BCSA.

**FIGURE 2:**
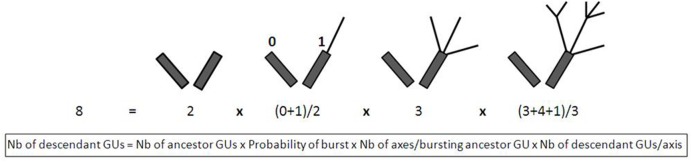
**Example of schematic representation of the four basic variables determining the total number of GUs produced during a growing cycle.** The ancestor GUs are represented by dark gray rectangles and the descendant GUs by black lines. Leaves are not represented. Quantification of each variable is noted below the schemes.

The number of descendant GUs can be broken down into basic variables that quantify chronologically ordered basic events. For a set of ancestor GUs, the total number of descendant GUs produced during a cycle can be described by the following equation (**Figure 2**).

$$\begin{array}{cc} \; & Number\,\;of\,\;descendant\,\;GUs\\ = & Number\,\;of\,\;ancestor\,GUs\\ \times & \mathrm{Pr}obality\,\;of\,\;burst\,\;of\,\;the\,\;ancestor\,\;GUs\\ \times & Number\,\;of\,\;axes\,\;per\,\;bursting\,\;ancestor\,\;GUs\\ \times & Number\,\;of\,\;descendant\,\;GUs\,\;per\,\;axis. \end{array}$$

These basic variables, i.e., the probability of burst (corresponding to the number of ancestor GUs that produced at least one descendant GU divided by the number of ancestor GUs), the number of axes per bursting ancestor GU, and the number of descendant GUs per axis, were used to analyze the setup of vegetative growth and to identify which step(s) was (were) affected by reproduction of the ancestor GU. One of our variables of interest, the number of terminal GUs, could be calculated in this way by changing the last term of the equation by the number of terminal descendant GUs per axis. Since this variable represented only one part of the total number of descendant GUs produced on an axis and since this proportion depended on the way the axis grew and branched, we preferred to consider the total number of descendant GUs for this specific analysis. We verified that the total number of descendant GUs and the number of terminal descendant GUs were positively correlated for the four cultivars (data not shown). The same equation was used for the second variable of interest, the leaf area produced per ancestor GU, after replacing the last term by the leaf area produced per axis.

Since it was difficult to precisely quantify the reproductive effort of an ancestor GU during a cycle (R1), the fate of the ancestor GU was considered as a gradient of reproductive effort, from vegetative (V, no cost of reproduction), to flowering (F, intermediate costs of reproduction) and fruiting (FR, high costs of reproduction). The concept of the costs of reproduction refers to the reproductive success in terms of seed production (Obeso, 2002). Since the mango fruit contains a single seed, reproduction during the following cycle (R2) was assessed by the number of fruits produced by the terminal GUs of an ancestor GU. At the scale of the scaffold branch, the reproductive effort during the first growing cycle (R1) and reproduction during the following cycle (R2) were quantified by the number of fruits produced by a scaffold branch, normalized by its size, i.e., divided by BCSA.

Data were analyzed for each cultivar and each cycle at the scales of the ancestor GU and of the scaffold branch. Analyses were carried out in three complementary steps (**Figure 1**):

(1) The effects of reproductive effort during a cycle (R1) on reproduction during the following cycle (R2) (R1→R2: **Figure 1A**, arrows 1).
(2) The effects of reproductive effort during a cycle (R1) on vegetative growth during the following cycle (V) (R1→V: **Figure 1A**, arrows 2).
(3) The effects of vegetative growth (V) on reproduction during the same cycle (R2) (V→R2: **Figure 1A**, arrows 3).

The variables recorded at the scale of the ancestor GUs were not Gaussian, and generalized linear models (GLMs) were used to test the effects of the fate of the ancestor GU on the vegetative and reproductive variables. A binomial distribution was used for binary response variables, and a Poisson or quasi-Poisson distribution was used for count response variables. Leaf area followed a Gaussian distribution after log-transformation, and analysis of variance was used to analyze the effect of the fate of ancestor GUs on this variable. Linear models (LM) were used to study relationships at the scale of the scaffold branch. A Tukey *post hoc* test procedure for GLM and LM was used for comparison of means with a significance level of *P* < 0.05. Statistical analyses were performed with R software (R Development Core Team, 2014), with the ‘multcomp’ and ‘MASS’ packages.

## Results

The magnitude of TCSA increase was between 2 and 3.1 over the three cycles depending on the cultivar (**Table 1**). The mean number of fruits produced per tree significantly increased between cycle 0 and cycle 1 and was similar for cycles 1 and 2 for Cogshall and Kensington Pride. It was stable across the three cycles for Irwin and José. The individual fruit mass was significantly different across cultivars, with Cogshall having heavy fruits and José light fruits. The number of fruits per fruiting ancestor GU was significantly higher for Irwin and José than for Kensington Pride and Cogshall.

**Table 1:** Trunk cross-sectional area (TCSA), normalized number of fruits, individual fruit mass and number of fruits per fruiting ancestor growth unit (GU) (mean ± SD) of four mango cultivars and three consecutive growing cycles.

Cultivar	TCSA (cm^2^)				Normalized number of fruits (nb/cm^2^)				Individual fruit mass (g)	Number of fruits per fruiting ancestor GU
	Cycle 0	Cycle 1	Cycle 2	*P*-value	Cycle 0	Cycle 1	Cycle 2	*P*-value		
Cogshall	40.4 ± 1.8 C, b	64.5 ± 2.5 B, b	85.4 ± 6.8 A, b	**<0.001**	0.4 ± 0.2 B	1.6 ± 0.2 A	1.6 ± 0.1 A, a	**<0.001**	410.6 ± 104.1 a	1.3 ± 0.6 c
Irwin	32.0 ± 5.8 B, bc	45.8 ± 7.9 B, c	67.3 ± 10.2 A, b	**0.005**	0.9 ± 0.2	1.3 ± 0.4	1.1 ± 0.1 ab	0.22	337.9 ± 93.9 c	2.2 ± 1.6 a
José	24.3 ± 4.2 C, c	44.8 ± 5.1 B, c	74.6 ± 10.4 A, b	**<0.001**	1.2 ± 0.4	1.4 ± 0.9	0.7 ± 0.5 b	0.43	210.4 ± 44.1 d	2.2 ± 1.4 a
Kensington Pride	56.1 ± 5.5 B, a	98.6 ± 4.5 B, a	142.6 ± 1.7 A, a	**<0.001**	0.7 ± 0.2 B	1.7 ± 0.1 A	1.3 ± 0.1 AB, ab	**0.01**	364.7 ± 92.0 b	1.5 ± 0.8 b
*P*-value	**<0.001**	**<0.001**	**<0.001**		0.07	0.84	**0.02**		**<0.001**	**<0.001**

*Individual fruit mass and the number of fruits per fruiting GU are averaged over the first two cycles. For the TCSA and the normalized number of fruits, means followed by different upper case letters are significantly different between cycles for the same cultivar, and means followed by lower case letters are significantly different between cultivars for the same cycle (analysis of variance followed by Tukey’s test). For the individual fruit mass and the number of fruits per fruiting ancestor GU, means followed by different lower case letters are significantly different between cultivars (analysis of variance followed by Tukey’s test). P-values in bold indicate significant tests (P < 0.05)*.

### Effects of Reproduction during One Cycle on Reproduction during the Following Cycle (R1→R2)

At the scale of the ancestor GU, the number of fruits produced by the descendant GUs was generally higher for the V ancestor GUs than for the FR ancestor GUs, but with differences between cultivars and cycles (**Table 2**). For Cogshall, descendant GUs from V ancestor GUs produced more fruits than those from F ancestor GUs during cycle 1. During cycle 2, the number of fruits produced by the descendant GUs was not affected by the reproductive effort of the ancestor GU. For Irwin, the reproductive effort of the ancestor GU did not affect the number of fruits produced during both cycles. For José, descendant GUs from V ancestor GUs produced more fruits than those from F ancestor GUs during cycle 1 and than those from F and FR ancestor GUs during cycle 2. Descendant GUs from F and FR ancestor GUs did not produce fruit during cycle 2. For Kensington Pride, the number of fruits was higher for descendant GUs from V ancestor GUs than those from F and FR ancestor GUs during cycle 1. The reproductive effort of the ancestor GU had no effect on the number of fruits produced during cycle 2.

**Table 2:** Effect of the reproductive effort of the ancestor GU [vegetative (V) < flowering (F) < fruiting (FR)] on the number of fruits (mean ± SD) produced by the descendant GUs of each ancestor GU during the following cycle, for four mango cultivars and two growing cycles.

Cycle	Cultivar	Fate of ancestor GU			*P*-value
		*V*	*F*	*FR*	
	Cogshall	2.2 ± 2.3 a	0.9 ± 1.1 b	-	**<0.001**
1	Irwin	-	1.6 ± 3.2	3.2 ± 3.3	0.07
	José	2.5 ± 4.1 a	0.9 ± 1.5 b	1.3 ± 2.0 ab	**0.01**
	Kensington Pride	8.8 ± 6.0 a	2.8 ± 2.5 b	1.8 ± 1.4 c	**<0.001**

	Cogshall	0.9 ± 1.7	0.8 ± 1.1	0.7 ± 1.0	0.42
2	Irwin	1.0 ± 1.5	0.9 ± 1.1	1.3 ± 1.8	0.22
	José	1.2 ± 2.0 a	0.0 ± 0.0 b	0.0 ± 0.0 b	**0.001**
	Kensington Pride	1.5 ± 2.2	1.1 ± 1.7	1.2 ± 1.4	0.41

*For each cycle and cultivar, means followed by different letters are significantly different (generalized linear model followed by Tukey’s test with P < 0.05). P-values in bold indicate significant tests (P < 0.05). Means are not computed when sample size is less than 5 (‘-’).*

At the scaffold branch scale, the number of fruits produced by Cogshall during cycle 1 was negatively related to the number of fruits produced during cycle 0 (*r*^2^ = 0.45, *P* < 0.001, *n* = 19; **Supplementary Figure S1**), and this relationship was positive between cycles 1 and 2 (*r*^2^ = 0.32, *P* = 0.007, *n* = 19; **Supplementary Figure S1**). No significant relationships were observed for the other cultivars.

### Effects of Reproduction during One Cycle on Vegetative Growth during the Following Cycle (R1→V)

**FIGURE 3:**
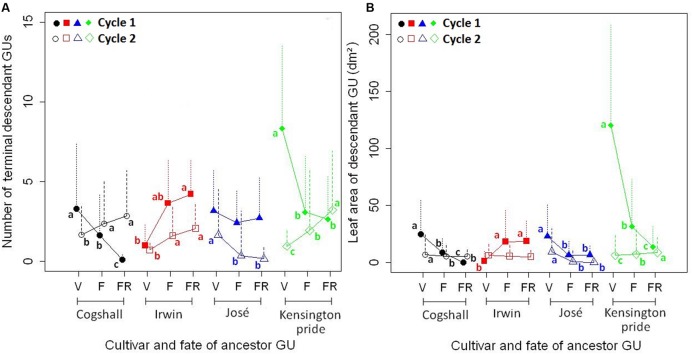
**R1→V relationships at the ancestor GU scale.** Effects of the reproductive effort of the ancestor GU [vegetative (V) < flowering (F) < fruiting (FR)] on the number of terminal descendant GUs **(A)** and on the leaf area of descendant GUs **(B)** produced during the following cycle (mean + SD) for four mango cultivars, Cogshall, Irwin, José, and Kensington Pride, and two growing cycles. For a given cultivar and cycle, means with different letters are significantly different [generalized linear model **(A)** and analysis of variance on log-transformed data **(B)**, followed by Tukey’s test].

The reproductive effort of the ancestor GU affected vegetative growth during the following cycle, i.e., the number of terminal GUs and the leaf area of descendant GUs, for the four cultivars and the two cycles (**Figure 3**). For Cogshall and Kensington Pride, F and FR ancestor GUs produced significantly less terminal GUs than V ancestor GUs during cycle 1, whereas they produced significantly more terminal GUs than V ancestor GUs during cycle 2 (**Figure 3A**). For Irwin, the reproductive effort of the ancestor GU affected the number of terminal GUs in the same way during both cycles: F and FR ancestor GUs produced more terminal GUs than V ancestor GUs. The opposite trend was observed for José: V ancestor GUs produced more terminal GUs than F and FR ancestor GUs. The differences were significant during cycle 2 but not during cycle 1.

The range of leaf area produced per ancestor GU was low for Irwin (0.35–69.9 dm^2^) intermediate for Cogshall (0.0–125.8 dm^2^) and José (0.0–131.1 dm^2^), and large for Kensington Pride (0.0–291.9 dm^2^) during cycle 1. For Cogshall and José, V ancestor GUs produced a larger leaf area during the following cycle than F and FR ancestor GUs during both cycles (**Figure 3B**). For Irwin, F and FR ancestor GUs produced a larger leaf area than V ancestor GUs during cycle 1, and no significant relationship was revealed during cycle 2. For Kensington Pride, V ancestor GUs produced four times more leaf area than F ancestor GUs, and nine times more than FR ancestor GUs during cycle 1. The opposite was observed during cycle 2, but with less absolute difference than during cycle 1.

The reproductive effort of the ancestor GU affected basic variables that characterize the processes involved in vegetative growth setup, i.e., the probability of burst of the ancestor GU, the number of axes produced per bursting ancestor GU, the number of descendant GUs per axis, and the leaf area of descendant GUs per axis, with an effect of cultivar and cycle (**Table 3**). For Irwin, the probability of burst was higher for F and FR than for V ancestor GUs during both cycles. In contrast, it was higher for V ancestor GUs during both cycles for José. For Cogshall and Kensington Pride, the effect of reproductive effort on the probability of burst differed between the two cycles. During cycle 1, it was high for the V and F ancestor GUs and low for the FR ancestor GUs, whereas it was higher for F and FR ancestor GUs during cycle 2. The number of axes produced per bursting ancestor GU was generally high for the FR ancestor GUs, low for the V ancestor GUs, and intermediate for the F ancestor GUs, except for Irwin and Cogshall during cycle 1 and José during cycle 2 where the relationships were not significant. The number of descendant GUs per axis was generally higher for V ancestor GUs than for F and FR ancestor GUs, except for Irwin where the number of descendant GUs per axis was higher for F ancestor GUs during cycle 1, and for V and F ancestor GUs during cycle 2. Similarly, the leaf area per axis was generally high for V ancestor GUs, low for FR ancestor GUs, and intermediate for F ancestor GUs, but with non-significant differences between F and FR ancestor GUs for José during cycle 2 and Irwin during cycle 1.

**Table 3:** Effect of the reproductive effort of the ancestor GU [vegetative (V) < flowering (F) < fruiting (FR)] on its vegetative growth during the following cycle, characterized by four variables (mean ± SD): probability of burst, number of axes per bursting ancestor GU, number of descendant GUs per axis, and leaf area of descendant GUs per axis for four mango cultivars and two growing cycles.

Cycle	Cultivar	Variable	Fate of ancestor GU			*P*-value
			*V*	*F*	*FR*	
	Cogshall	Ancestor probability of burst	0.7 ± 0.5 a	0.6 ± 0.5 a	0.1 ± 0.3 b	**<0.001**
		Number of axes/ancestor	2.5 ± 1.5	2.5 ± 1.3	-	0.4
		Number of GU/axis	3.3 ± 2.9 a	1.8 ± 1.3 b	-	**<0.001**
		Leaf area/axis (dm^2^)	14.6 ± 14.3 a	6.2 ± 6.2 b	2.2 ± 0.3 b	**<0.001**
	
	Irwin	Ancestor probability of burst	0.4 ± 0.6 b	1.0 ± 0.0 a	1.0 ± 0.0 a	**<0.001**
		Number of axes/ancestor	-	3.5 ± 2.8	4.2 ± 2.2	0.22
		Number of GU/axis	1.9 ± 0.9 b	2.3 ± 1.2 a	1.7 ± 0.6 b	**<0.001**
1		Leaf area/axis (dm^2^)	6.1 ± 4.6 a	5.3 ± 4.6 ab	4.5 ± 4.1 b	**<0.001**
	
	José	Ancestor probability of burst	0.9 ± 0.9 a	0.7 ± 0.5 b	0.8 ± 0.4 ab	**0.009**
		Number of axes/ancestor	1.9 ± 1.3 b	3.4 ± 2.0 a	3.5 ± 2.7 a	**<0.001**
		Number of GU/axis	3.0 ± 2.5 a	1.1 ± 0.4 b	1.1 ± 0.6 b	**<0.001**
		Leaf area/axis (dm^2^)	12.3 ± 12.2 a	2.7 ± 2.0 b	1.1 ± 3.2 c	**<0.001**
	
	Kensington Pride	Ancestor probability of burst	1.0 ± 0.0 a	0.9 ± 0.3 a	0.7 ± 0.5 b	**<0.001**
		Number of axes/ancestor	2.2 ± 1.6 b	3.0 ± 1.7 ab	3.5 ± 2.1 a	**0.03**
		Number of GU/axis	6.3 ± 4.2 a	1.9 ± 1.3 b	1.4 ± 1.4 c	**<0.001**
		Leaf area/axis (dm^2^)	54.2 ± 23.1 a	12.0 ± 12.3 b	6.2 ± 9.8 c	**<0.001**

	Cogshall	Ancestor probability of burst	0.5 ± 0.5 b	0.6 ± 0.5 a	0.7 ± 0.5 a	**<0.001**
		Number of axes/ancestor	2.1 ± 1.4 c	3.5 ± 1.8 b	4.3 ± 2.5 a	**<0.001**
		Number of GU/axis	2.5 ± 2.6 a	1.3 ± 1.1 b	1.0 ± 0.3 c	**<0.001**
		Leaf area/axis (dm^2^)	7.5 ± 10.7 a	2.3 ± 3.0 b	2.1 ± 1.5 c	**<0.001**
	
	Irwin	Ancestor probability of burst	0.6 ± 0.5 b	0.7 ± 0.4 a	0.8 ± 0.4 a	**<0.001**
		Number of axes/ancestor	1.2 ± 0.6 b	2.2 ± 1.0 a	2.7 ± 1.4 a	**<0.001**
		Number of GU/axis	2.0 ± 0.9 a	1.8 ± 0.9 a	1.1 ± 0.4 b	**<0.001**
2		Leaf area/axis (dm^2^)	10.4 ± 10.8 a	3.6 ± 3.0 b	2.5 ± 2.1 c	**<0.001**
	
	José	Ancestor probability of burst	0.5 ± 0.5 a	0.2 ± 0.4 b	0.1 ± 0.3 b	**<0.001**
		Number of axes/ancestor	2.4 ± 1.9	2.0 ± 1.4	1.6 ± 1.1	0.29
		Number of GU/axis	2.1 ± 1.4 a	1.2 ± 1.0 b	1.3 ± 0.3 ab	**<0.001**
		Leaf area/axis (dm^2^)	8.5 ± 6.6 a	4.4 ± 3.1 b	3.8 ± 2.4 b	**<0.001**
	Kensington Pride	Ancestor probability of burst	0.2 ± 0.4 b	0.5 ± 0.5 a	0.5 ± 0.4 a	**<0.001**
		Number of axes/ancestor	2.3 ± 1.5 c	3.9 ± 2.2 b	5.9 ± 3.2 a	**<0.001**
		Number of GU/axis	2.8 ± 2.6 a	1.2 ± 0.9 b	1.1 ± 0.6 c	**<0.001**
		Leaf area/axis (dm^2^)	13.8 ± 15.6 a	4.2 ± 4.7 b	2.9 ± 3.3 c	**<0.001**

*In the same line, means followed by different letters are significantly different (generalized linear model, and analysis of variance on log-transformed data for the leaf area per axis, followed by Tukey’s test). P-values in bold indicate significant tests (P < 0.05). Means are not computed when sample size is less than 5 (‘-‘)*.

**FIGURE 4:**
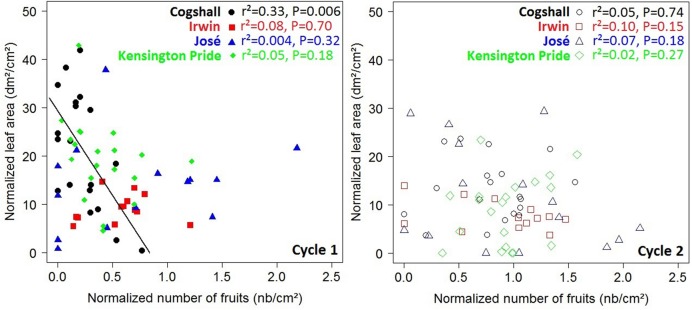
**R1→V relationships at the scaffold branch scale.** Relationships between the normalized number of fruits produced during the previous cycle and the normalized leaf area produced by descendant GUs during the current cycle at the scaffold branch scale for four mango cultivars, Cogshall, Irwin, José, and Kensington Pride, and two growing cycles. Coefficient of determination (*r*^2^) and *P*-value associated with the linear adjustments are given in the figure. Regression lines are presented for significant relationships (*P* < 0.05).

At the scaffold branch scale, a negative relationship was revealed for Cogshall between the number of fruits produced during cycle 0 and the leaf area of descendant GUs produced during cycle 1 (**Figure 4**). No significant relationship was observed between these variables for the other cultivars and for any of the cultivars between cycles 1 and 2 (**Figure 4**). The number of terminal GUs produced during cycle 1 was negatively related to the number of fruits produced during cycle 0 for Cogshall (*r*^2^ = 0.41, *P* = 0.002, *n* = 19). This relationship was positive for José (*r*^2^ = 0.39, *P* = 0.007, *n* = 16). Positive relationships were shown between cycles 1 and 2 for Cogshall (*r*^2^ = 0.25, *P* = 0.02, *n* = 19) and for Irwin (*r*^2^ = 0.39, *P* = 0.01, *n* = 13) (**Supplementary Figure S2**). No significant relationship was revealed between these variables for Kensington Pride during either cycle.

### Effects of Vegetative Growth on Reproduction during the Same Cycle (V→R2)

**FIGURE 5:**
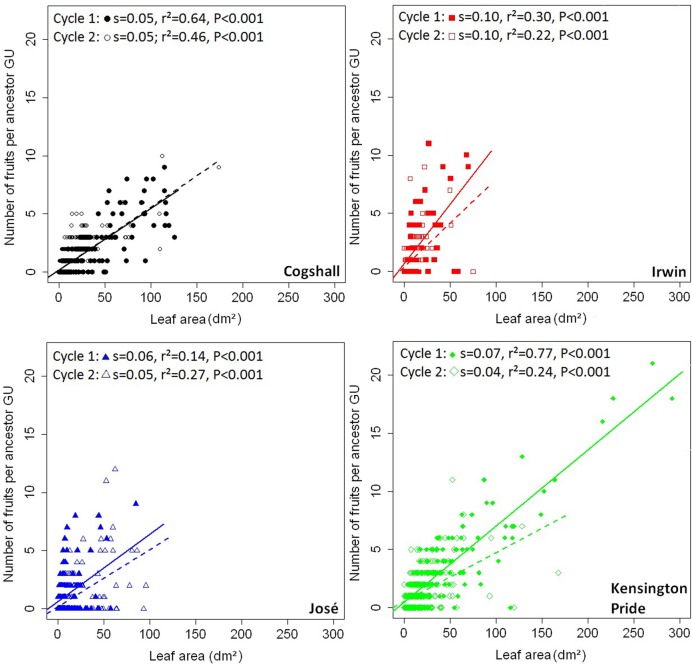
**V→R2 relationships at the ancestor GU scale.** Relationships between leaf area of descendant GUs produced by each ancestor GU and the number of fruits produced by these descendant GUs for four mango cultivars, Cogshall, Irwin, José, and Kensington Pride, and two growing cycles. Slope (s), coefficient of determination (*r*^2^) and *P*-value associated with the linear adjustments (cycle 1: solid line; cycle 2: dotted line) are given for each cycle.

At the scale of the ancestor GU, positive relationships were shown between the leaf area of descendant GUs and the number of fruits produced by these descendant GUs for all cultivars and during both cycles (**Figure 5**). These relationships were satisfactorily approximated by linear regressions. For each cycle, slopes of the relationships were significantly different between cultivars (analysis of covariance, *P* < 0.001). Slopes were significantly higher for Irwin and Kensington Pride than for Cogshall during cycle 1, and were higher for Irwin than for Kensington Pride during cycle 2.

Positive relationships were also revealed between the number of terminal GUs and the number of fruits they produced for all cultivars and during both cycles (**Supplementary Figure S3**). Slopes of the relationships were significantly different between cultivars for both cycles (analysis of covariance, *P* < 0.001). Slopes were higher for Kensington Pride and Irwin than for Cogshall and José during cycle 1, and were higher for Irwin than Cogshall, Kensington Pride, and José during cycle 2.

**FIGURE 6:**
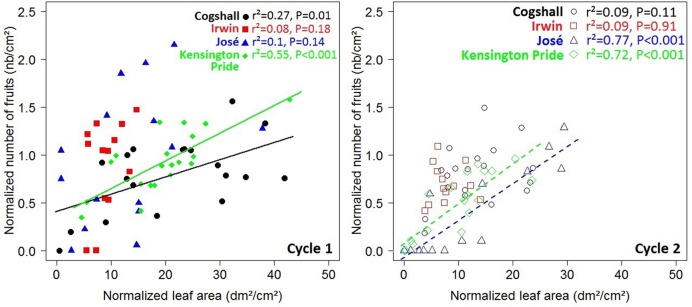
**V→R2 relationships at the scaffold branch scale.** Relationships between the leaf area produced during a cycle and the number of fruits produced during this cycle at the scaffold branch scale for four mango cultivars, Cogshall, Irwin, José, and Kensington Pride, and two growing cycles. Coefficient of determination (*r*^2^) and *P*-value associated with the linear adjustments are given in the figure. Regression lines are presented for significant relationships (*P* < 0.05).

At the scaffold branch scale, positive relationships were observed between the leaf area of descendant GUs and the number of fruits produced by these descendant GUs for Cogshall and Kensington Pride during cycle 1, and for José and Kensington Pride during cycle 2 (**Figure 6**). No significant relationship was observed for Irwin. Positive relationships were revealed between the number of terminal GUs and the number of fruits they produced during cycle 1 for Cogshall (*r*^2^ = 0.49, *P* < 0.001, *n* = 19) and Kensington Pride (*r*^2^ = 0.26, *P* = 0.01, *n* = 20), and during cycle 2 for Cogshall (*r*^2^ = 0.24, *P* = 0.02, *n* = 19) and Irwin (*r*^2^ = 0.46, *P* = 0.007, *n* = 13; **Supplementary Figure S4**). No relationship was observed for José.

## Discussion

Constraining relationships link life history traits, i.e., survival, growth and reproduction, and lead to compromises, or costs, for plants (Reznick, 1985). Some researchers have addressed the costs of reproduction by considering reproduction (R) and vegetative growth (V), and by alternatively analyzing the effects of reproduction during one cycle on reproduction during the following cycle (R→R) (Jonkers, 1979; Monselise and Goldschmidt, 1982; Kawamura and Takeda, 2006; Muñoz-Fambuena et al., 2011), and/or the effects of reproduction on vegetative growth (R→V) (Miyazaki et al., 2002; Kawamura and Takeda, 2006; Żywiec and Zielonka, 2013). Some of these studies use plant manipulation such as stem girdling (Newell, 1991) or the experimental reduction or increase of fruit production (Ashman, 1992; Euler et al., 2012; Toivonen and Mutikainen, 2012; Sletvold and Ågren, 2015). In our study, we characterized, without any plant manipulation, the effects of reproduction during one cycle on reproduction during the following cycle (R1→R2), and further broke down this global effect considering two structural and temporal steps, namely the effects of reproduction on vegetative growth during the following cycle (R1→V), and the effects of vegetative growth on the subsequent reproduction (V→R2).

Our study did not take the effect of the growing cycle into account for two reasons. First, environmental factors were different between the two cycles (especially rainfall during vegetative growth after harvest; data not shown), and plant development and reproduction are affected by external factors such as temperature, rainfall and light radiation. Second, the trees were young and underwent ontogenic changes (Thomas and Winner, 2002; Barthélémy and Caraglio, 2007), e.g., a decrease in vegetative growth associated to an increase of fruit production (Dambreville et al., 2013) that might have affected the observed relationships between the two cycles.

### Deciphering the Delayed Costs of Reproduction

Delayed costs of reproduction, i.e., from one cycle to the following, differed between cultivars and between scales. In the following, our results are discussed by scale, first at the ancestor GU scale and then at the scaffold branch scale.

#### Scale of the Ancestor GU

**FIGURE 7:**
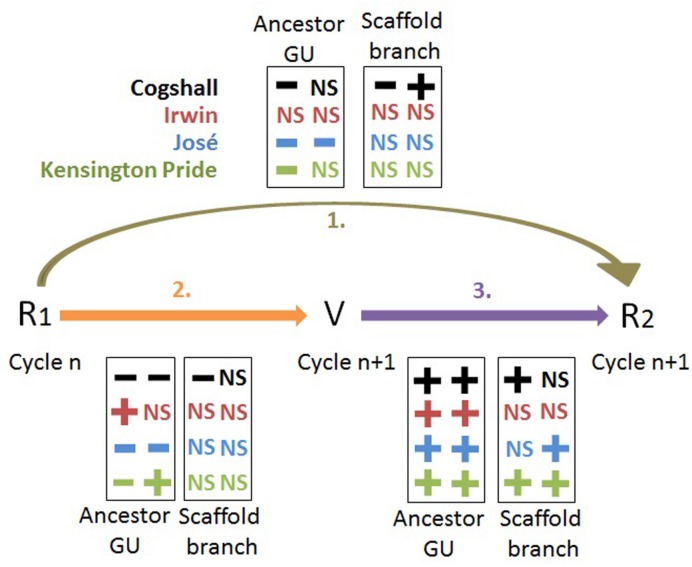
**Synthesis of the R1→V→R2 relationships.** Relationships between reproduction (R1, R2) and vegetative growth (V), expressed as the leaf area of descendant GUs, for four mango cultivars at the scales of the ancestor GU and of the scaffold branch. The ‘**+**’ and ‘**-**’ symbols represent significant positive and negative relationships, respectively (*P* < 0.05), and NS indicates non-significant relationships. The first and second columns in each box represent the results from cycle 0 to cycle 1, and from cycle 1 to cycle 2, respectively, for R1→R2 and R1→V, and within cycle 1 and cycle 2, respectively, for the V→R2 relationships.

The effect of the reproductive effort on vegetative growth differed between cultivars (**Figure 7**). Cogshall and José showed a negative effect during both cycles, Irwin showed a positive effect during the first cycle and no effect during the second cycle, and Kensington Pride showed opposite effects, negative during the first cycle and positive during the second cycle. The negative R1→V relationships indicated delayed costs of reproduction on vegetative growth at the ancestor GU scale. These costs were high for Cogshall and José, null for Irwin and intermediate for Kensington Pride. Previous studies have also shown negative R→V relationships at the shoot scale, e.g., the inhibition of shoot emergence by fruit growth (Plummer, 1987; Davenport, 1990; Lovatt, 2010; Smith and Samach, 2013), or the reduction of leaf length on reproductive shoots (Tuomi et al., 1982). The reproductive fate of *Vaccinium hirtum* branches negatively affects their number of shoots (Kawamura and Takeda, 2006). In contrast, pistachio shoot growth is not affected by fruit production (Stevenson et al., 2000). In our study, the decomposition of vegetative growth into four basic variables that quantify chronologically ordered basic events allowed a better understanding of the effects of reproduction on vegetative growth.

The similarity between the probability of burst of the ancestor GU, on the one hand (**Table 3**), and the effects of the reproductive effort of the ancestor GU on the leaf area of the descendants GUs on the other (**Figure 3B**), suggests that the probability of burst was an important factor in determining vegetative growth. The effect of the reproductive effort on the probability of burst was negative for José and positive for Irwin. Kensington Pride and Cogshall showed opposite effects, negative during the first cycle and positive during the second cycle. The observed negative effect could be related to two non-exclusive mechanisms that have been proposed to better understand the mechanism of fruit dominance on vegetative growth (Smith and Samach, 2013). The first one is a trophic mechanism, a competition for resources between reproduction and vegetative outgrowth. Inflorescence growth, which represents small sinks over a relatively short time, about 3–4 weeks, and fruit growth, which represents strong sinks over a long time, about 4 months, would lead to local carbohydrate depletion in the reproductive ancestor GUs, hindering vegetative outgrowth. This depletion persists after removal of the sinks since a delay is needed to recover a satisfactorily local carbohydrate status (Wardlaw, 1990; Spann et al., 2008). The second mechanism is related to hormonal signaling. Inflorescences and fruits produce hormones, in particular, auxin and cytokinins (Sandip et al., 2015). Several studies have shown that the basipetal auxin flow from the growing fruit inhibits the auxin flow out of the axillary buds (Waldie et al., 2010; Domagalska and Leyser, 2011; Smith and Samach, 2013) and, consequently, prevents shoot outgrowth (Ferguson and Beveridge, 2009).

Focusing on significant relationships, it can be observed that the number of axes per GU was higher for fruiting ancestor GUs than for vegetative ancestor GUs for all cultivars and during both cycles (**Table 3**). This result was probably the consequence of the loss of apical control of the reproductive ancestor GUs for which the apical bud turned into an inflorescence. Apical control is defined by Wilson (2000) as the inhibition of lateral bud outgrowth by the apical bud. When the inflorescence or the fruit are no longer present on the shoot, the inhibitory effect of reproduction on bud outgrowth disappears (see previously). The outgrowth of lateral buds then occurs without apical bud and therefore without apical control, enabling the outgrowth of more lateral buds. In contrast, vegetative GUs have an active and permanent apical bud that is able to inhibit or limit the outgrowth of lateral buds (Normand et al., 2009; Waldie et al., 2010).

An increase in the reproductive effort of the ancestor GU leads to a decrease in the number and the leaf area of descendant GUs per axis. This could be due to the combination of two factors, one temporal and one architectural. The first one is linked to phenology. Vegetative growth is delayed on reproductive ancestor GUs (Dambreville et al., 2013). The second factor is an architectural factor. A vegetative ancestor GU produces one apical GU, and possibly one or few lateral GUs. Apical GUs have larger leaf area and are able to branch more than lateral GUs (Normand et al., 2009). An endogenous rhythm of 2 months between the burst of successive GUs has been detected in mango (Anwar et al., 2011; Dambreville et al., 2013). Consequently, vegetative ancestor GUs burst earlier and their descendant GUs can grow and branch one or several times during the vegetative growth season (**Figure 1**), thus resulting in more descendant GUs and a larger leaf area than reproductive ancestor GUs.

Various mechanisms of compensation often make it difficult to detect the costs of reproduction (Tuomi et al., 1983; Obeso, 2002). On the basis of our results and the literature, it is possible to propose three mechanisms of compensation at the ancestor GU scale. The first one is the loss of apical control on reproductive ancestor GUs, leading to a higher number of descendant axes than on vegetative ancestor GUs (**Table 3**). The second one is the increased photosynthesis in leaves near growing mango fruits (Urban et al., 2003, 2004), leading to a slower local depletion of carbohydrates. The third one is the timing of reproduction and vegetative growth. A large part of vegetative growth occurs after harvest (**Figure 1**), therefore limiting direct competition with reproduction. These mechanisms could probably explain the non-significant or even positive R1→V relationships for Irwin and Kensington Pride (**Figure 7**). Despite these mechanisms, significant costs of reproduction on vegetative growth have been shown for José, Cogshall, and Kensington Pride.

Vegetative growth had a linear and positive effect on reproduction (V→R2) for all cultivars and during both cycles (**Figures 5** and **7**; **Supplementary Figure S2**). A positive and linear relationship has been similarly demonstrated between plant size and reproduction in *Arum italicum* (Méndez and Obeso, 1993). In ‘Hass’ avocado, vegetative growth is a key point enabling return bloom and fruit production (Lovatt, 2010). Since floral induction of mango occurs just before the burst of inflorescences (Davenport, 2009), and is therefore not affected by growing fruits of the previous cycle, two non-exclusive hypotheses could be proposed to explain our results. The first one is that a large leaf area stimulates flowering and fruiting for trophic reasons and also via the production of a florigenic promoter, detailed later in the discussion. The second hypothesis is related to demography. More terminal GUs means more potential fruiting sites and, potentially, more fruits. The linear relationships (**Figure 5**) suggested that the probability of fruiting was constant and independent of vegetative growth; otherwise, relationships would be non-linear. Their slopes differed between cultivars. A high slope indicated that a given increase in vegetative growth corresponded to a larger increase in the number of fruits produced compared to a lower slope. It denoted a kind of efficiency of vegetative growth to produce fruits. For example, Irwin showed higher slopes than the other cultivars during both cycles, and was able to produce as many fruits as the other cultivars with limited vegetative growth.

The R1→R2 relationships are a combination of the R1→V and V→R2 relationships. They are generally negative, but with a marked cultivar effect (**Figure 7**). These negative relationships indicate delayed costs of reproduction on the subsequent reproduction. Three groups of cultivars could be distinguished: Irwin with no significant R1→R2 effect during both cycles, José with negative effects during both cycles, and Cogshall and Kensington Pride with a negative effect during one cycle and no effect during the other cycle. Consequently, the costs of reproduction at the ancestor GU scale were high for José, null for Irwin, and intermediate for Cogshall and Kensington Pride. Previous studies revealed negative R→R relationships. Current reproduction of *Vaccinium hirtum* reduces future reproductive output at the shoot level through competition with vegetative growth (Kawamura and Takeda, 2006). The results of Cogshall and Kensington Pride suggested that the cycle could affect the costs of reproduction at the ancestor GU scale, possibly through the effects of environmental conditions. Although they were similar for all trees within a given cycle, they could differ from one cycle to the other. The costs of reproduction may be influenced by environmental conditions such as soil characteristics (Biere, 1995), water availability (Euler et al., 2012) and duration of growing season and altitude (Obeso, 2002). Biere (1995) showed that they are greater in poor sites than in fertile sites. The consistent presence or absence of the costs of reproduction during both cycles for José and Irwin, respectively, suggested that the environment did not affect the behavior of these cultivars. It may be expected that high tree fruit load leads to higher and, therefore, more detectable costs of reproduction at the ancestor GU scale. Mean tree fruit load was low during cycle 0 and high during cycle 1 for Cogshall and Kensington Pride (**Table 1**), whereas the costs of reproduction were significant following cycle 0, and non-significant following cycle 1 for both cultivars. Mean tree fruit load was similar for José and Irwin during both cycles, whereas the costs of reproduction were significant for José and non-significant for Irwin during both cycles. These results suggest that the costs of reproduction at the scale of the ancestor GU were not related to the mean tree fruit load and were consequently cultivar-dependent.

The fact that only negative or non-significant R1→R2 relationships were revealed at the ancestor GU scale suggested that the negative effects of reproduction on vegetative growth were predominant over the positive impact of vegetative growth on reproduction. The general trend was that the higher the reproductive effort was, the less the vegetative growth would be during the following cycle and, consequently, the less fruits produced by this vegetative growth. The cultivar effects on the costs of reproduction were then mainly determined by the R1→V relationships, in particular, on the probability of burst of the ancestor GU.

#### Scale of the Scaffold Branch

The effects of reproduction on vegetative growth (R1→V) were not significant at the scaffold branch scale, except for Cogshall during cycle 1. We could expect that the results revealed at the ancestor GU scale were also seen at the scale of the scaffold branch by aggregation. When studying relationships at the scaffold branch scale on data normalized by the scaffold branch cross-sectional area, we made the implicit assumption of the autonomy of the scaffold branches, independently of their size. The absence of a significant relationship at the scale of the scaffold branch therefore revealed a partial autonomy with respect to the processes underlying these effects (Sprugel et al., 1991; Obeso, 2002). This partial autonomy could be related to the exchanges of carbohydrates between scaffold branches or between scaffold branches and areas of carbohydrate storage such as the trunk and the roots. The allocation of carbohydrates to the growing mango fruits from other parts of the tree can be considerable (Stassen et al., 1997; Davie et al., 1999). The partial autonomy could then be interpreted as a mechanism of compensation of the costs of reproduction, interfering with their detection at the scaffold branch scale (Obeso, 2002). The other two compensatory mechanisms proposed at the scale of the ancestor GU, namely the increased photosynthesis and the timing of reproduction and vegetative growth, can be advanced at this scale too. Contrary to our results, competition between fruit load and subsequent vegetative development has been reported in apple where heavy fruit load decreases vegetative development (Jonkers, 1979; Forshey and Elfving, 1989) and secondary growth (Lauri et al., 2010) in the same year. Similarly, the increase in plant biomass was lower when more reproductive structures were produced in the previous year in *Carex secalina*, a perennial monoecious species (Bogdanowicz et al., 2011). Moreover, several studies have found that there are less new shoots during years of high fruit production than during years of low fruit production at the tree scale in pistachio (Weinbaum et al., 1994; Brown et al., 1995; Rosecrance et al., 1996; Picchioni et al., 1997).

In contrast, the relationships between vegetative growth and reproduction were significant at the scaffold branch scale for Kensington Pride during both cycles, Cogshall during cycle 1, and José during cycle 2, suggesting an autonomy of scaffold branches for the processes underlying flowering and fruiting, at least for these cultivars. Three processes could be proposed. The first one is at the branch scale and is related to shoot demography. Substantial vegetative growth means a high number of terminal GUs prone to flower and set fruit. The second process is at the scale of a group of GUs close to each other. In mango, a floral promoter is synthesized in the leaves and is able to move basipetaly and acropetaly in the phloem up to about 1 m to the potential flowering sites (Davenport et al., 2006; Ramírez and Davenport, 2010). The third process is at the scale of the ancestor GUs and is related to its architectural and temporal traits (date of birth, fate, apical or lateral position) (Dambreville et al., 2013) and to its morphology (Normand et al., 2009), which affect its ability to flower and set fruit. These processes are local, at the branch scale or at a smaller scale, therefore conferring branch autonomy with respect to these processes. The behavior at the scaffold branch scale is then the sum of the behavior of ancestor GUs. The number of fruits produced was not linked to vegetative growth at the scale of the scaffold branch during both cycles for Irwin, whereas it was the case at the ancestor GU scale. This result suggests a partial autonomy of Irwin branches with respect to the flowering and fruiting processes, with a more uniform allocation of carbohydrates to potential flowering points for example. Moreover, this cultivar showed a smaller range of leaf area at the scale of the scaffold branch compared to the other cultivars (**Figure 6**), making it more difficult to detect a significant relationship. Contrary to our results, negative relationships have been shown between leaf area and seed production in various species (Herben et al., 2012). Moreover, Hulshof et al. (2012) showed a negative relationship between the relative growth rate of above-ground biomass and biomass allocated to reproduction in *Bursera simaruba* (L.).

The R1→R2 relationships were a combination of the R1→V and V→R2 relationships at the scaffold branch scale. Those relationships were not significant, except for Cogshall. This probably resulted from the partial branch autonomy and the other mechanisms of compensation opposed to the negative effect of reproduction on vegetative growth (R1→V). Cogshall showed costs of reproduction at the scaffold branch scale during the first cycle. In contrast, a positive relationship between R1 and R2 was revealed during cycle 2, and could be related to terminal GUs demography. Cogshall produced a larger number of terminal GUs on fruiting branches during cycle 2 (**Supplementary Figure S2**), leading to a large fruit production (**Supplementary Figure S4**).

### Relationships between the Costs of Reproduction and Irregular Bearing

Our results showed a clear effect of the cultivar on the costs of reproduction. The costs of reproduction detected at the scale of the ancestor GU (**Figure 7**) discriminated three groups of cultivars: José with significant costs of reproduction during both cycles, Irwin with no cost of reproduction during both cycles, and Cogshall and Kensington Pride with costs of reproduction during cycle 1 and no cost of reproduction during cycle 2. These groups corresponded to the known fruiting pattern of these cultivars, with José being an irregular bearer, Irwin being a regular bearer and Kensington Pride and Cogshall being quite regular bearers (Campbell, 1992; Knight et al., 2009; Dambreville et al., 2014; unpublished data). However, the costs of reproduction could not be detected at the scaffold branch scale. The lack of detection of costs of reproduction at the scaffold branch scale does not imply that they do not occur at the tree scale (Obeso, 2002, 2004), which is the pertinent scale for studying irregular bearing.

Few studies have linked irregular bearing to the costs of reproduction. Pistachio, that, like the mango, belongs to the Anacardiaceae family, shows strong alternate bearing (Spann et al., 2008; Rosenstock et al., 2010). Stevenson and Shackel (1998) showed that alternate bearing corresponds to a switch in biomass partitioning between vegetative and reproductive growth. During the ‘off’ year, biomass is mainly allocated to vegetative growth rather than to fruit production.

### Directions for Future Research

The process of domestication has led to the selection of genotypes with a high potential for fruit production compared to wild genotypes. Those genotypes therefore allocate more resources to reproduction, which could lead to higher costs of reproduction. These costs could then be easier to detect in agricultural selected genotypes (Obeso, 2002). For wild long-lived species like trees, survival seems to be more important than reproduction in any given year (Crawley, 1985, 1997). Further investigations could be carried out on both cultivated mango cultivars with contrasted fruiting patterns and on wild mango genotypes placed in the same environment, in order to assess the effects of domestication on the costs of reproduction and associated compensatory mechanisms.

The costs of reproduction were not always detected in our study, even at the GU scale, suggesting different types of compensatory mechanisms at different scales, from the leaf to the whole tree. These mechanisms could be related to branch autonomy and to an increased efficiency of resource use (photosynthesis, carbohydrates and, possibly, water and nutrients). It would be interesting to study and better understand these mechanisms. This knowledge could be useful to identify relevant traits for the selection of regular bearer cultivars.

A complementary study could also be carried out to evaluate the costs of reproduction at the whole tree scale with an appropriate experimental design. Our experiment was designed to exhaustively capture data at the scales of the ancestor GUs and of the scaffold branches. This substantial experimental effort did not make it possible to record data on several trees per cultivar. The limited number of trees studied per cultivar (*n* = 3) was low compared to the large number of replications, e.g., 36 trees (Żywiec and Zielonka, 2013) or 60 trees (Obeso, 1997), generally used in studies addressing the measure of the costs of reproduction at the tree scale. Moreover, the costs of reproduction are often studied at the tree scale with manipulation, reduction or increase, of fruit production in order to maximize the range of reproductive effort (Obeso, 2002; Sletvold and Ågren, 2015), which was not the case in our experiment. In some species such as *Lathyrus vernus*, the costs of reproduction are only detectable after manipulation of the reproductive effort (Ehrlén and Van Groenendael, 2001).

## Conclusion

Studies on the costs of reproduction have been conducted on herbaceous perennial plants (Primack, 1979 on *Plantago* sp.; Primack and Stacy, 1998 on *Cypripedium acaule*) and on woody perennial plants (Cipollini and Stiles, 1991 on *Nyssa sylvatica*; Obeso, 1997 on *Ilex aquifolium*; Suzuki, 2000 on *Eurya japonica*). To the best of our knowledge, our study is the first to carry out a comprehensive analysis of the costs of reproduction on a cultivated tree species.

We have shown that, globally, reproduction during one cycle had a negative effect on reproduction during the following cycle in mango. We also showed that vegetative growth that occurs between the two reproductive events mitigated this negative effect. These effects occurred mainly at the GU scale, less frequently at the scaffold branch scale, and were clearly cultivar-dependent.

## Author Contributions

FN designed the study and acquired data. MC, P-EL, FN analyzed data, interpreted results, and wrote the paper. All of the authors read and approved the manuscript.

## Conflict of Interest Statement

The authors declare that the research was conducted in the absence of any commercial or financial relationships that could be construed as a potential conflict of interest.

## Supplementary material

FIGURE S1 **Relationships between the normalized number of fruits produced during the previous cycle and the normalized number of fruits produced during the current cycle at the scaffold branch scale for four mango cultivars, Cogshall, Irwin, José, and Kensington Pride, and two growing cycles.** The coefficient of determination (*r*^2^) and *P*-value associated with linear adjustments are given in the figure. Regression lines are presented for significant relationships (*P* < 0.05).

FIGURE S2 **Relationships between the normalized number of fruits produced during the previous cycle and the normalized number of terminal descendant growth units (GUs) produced during the current cycle at the scaffold branch scale for four mango cultivars, Cogshall, Irwin, José, and Kensington Pride, and two growing cycles.** The coefficient of determination (*r*^2^) and *P*-value associated with linear adjustments are given in the figure. Regression lines are presented for significant relationships (*P* < 0.05).

FIGURE S3 **Relationships between the number of terminal descendant GUs produced by each ancestor GU and the number of fruits produced by these terminal descendant GUs for four mango cultivars, Cogshall, Irwin, José, and Kensington Pride, and two growing cycles.** The slope (s), coefficient of determination (*r*^2^) and *P*-value associated with linear adjustments (cycle 1: solid line; cycle 2: dotted line) are given for each cycle.

FIGURE S4 **Relationships between the number of terminal descendant GUs produced during a cycle and the number of fruits produced during this cycle at the scaffold branch scale for four mango cultivars, Cogshall, Irwin, José, and Kensington Pride, and two growing cycles.** The coefficient of determination (*r*^2^) and *P*-value associated with the linear adjustments are given in the figure. Regression lines are presented for significant relationships (*P* < 0.05).
