# Supplementary figures and images for: Deciphering the Costs of Reproduction in Mango – Vegetative Growth Matters
Source: Front Plant Sci. 2016 Oct 21;7:1531. doi: 10.3389/fpls.2016.01531 (PMC5073132; doi:10.3389/fpls.2016.01531)

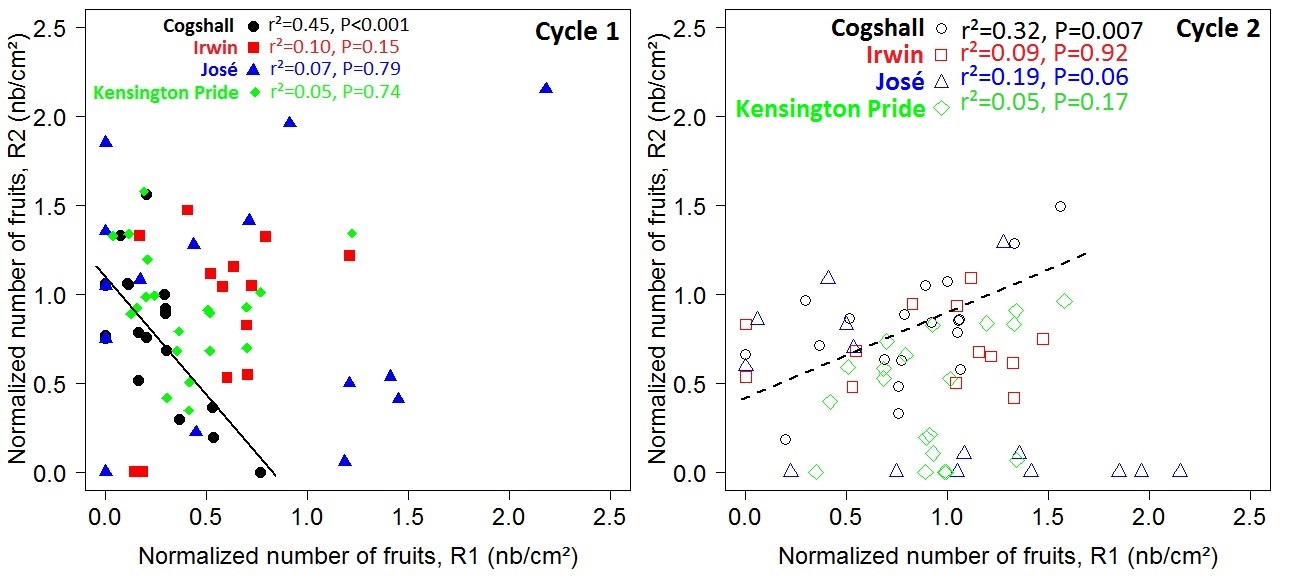

Supplement: FIGURE S1 — Relationships between the normalized number of fruits produced during the previous cycle and the normalized number of fruits produced during the current cycle at the scaffold branch scale for four mango cultivars, Cogshall, Irwin, José, and Kensington Pride, and two growing cycles. The coefficient of determination (r2) and P-value associated with linear adjustments are given in the figure. Regression lines are presented for significant relationships (P < 0.05). [file Image_1.jpg]

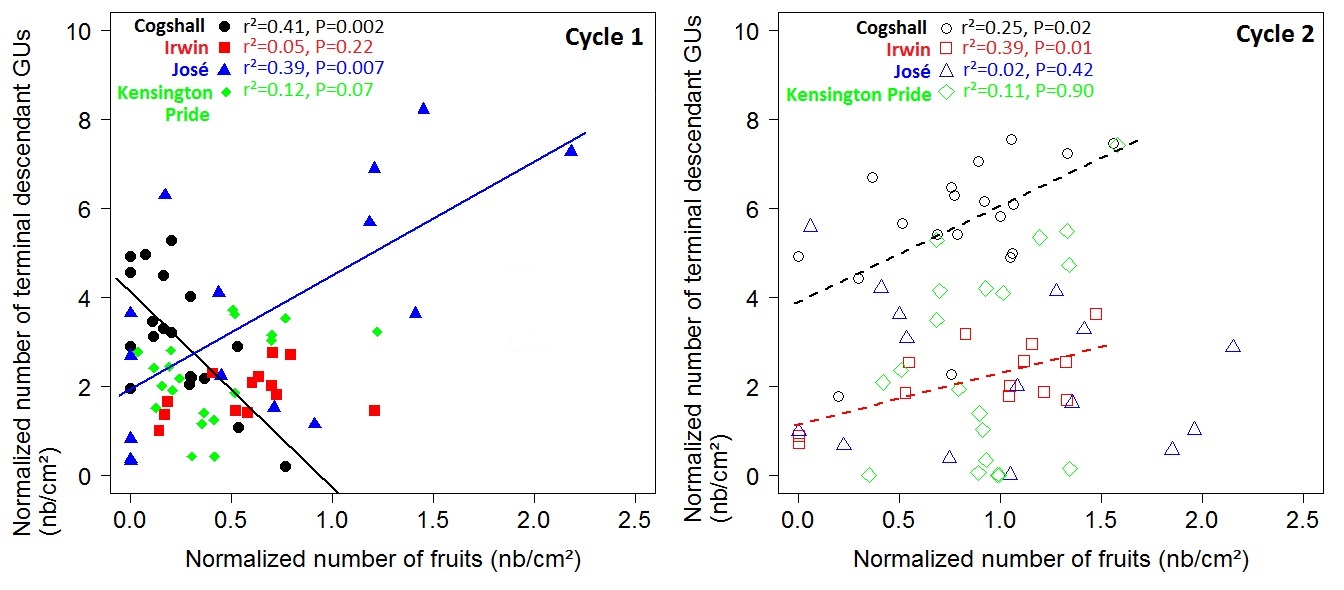

Supplement: FIGURE S2 — Relationships between the normalized number of fruits produced during the previous cycle and the normalized number of terminal descendant growth units (GUs) produced during the current cycle at the scaffold branch scale for four mango cultivars, Cogshall, Irwin, José, and Kensington Pride, and two growing cycles. The coefficient of determination (r2) and P-value associated with linear adjustments are given in the figure. Regression lines are presented for significant relationships (P < 0.05). [file Image_2.jpg]

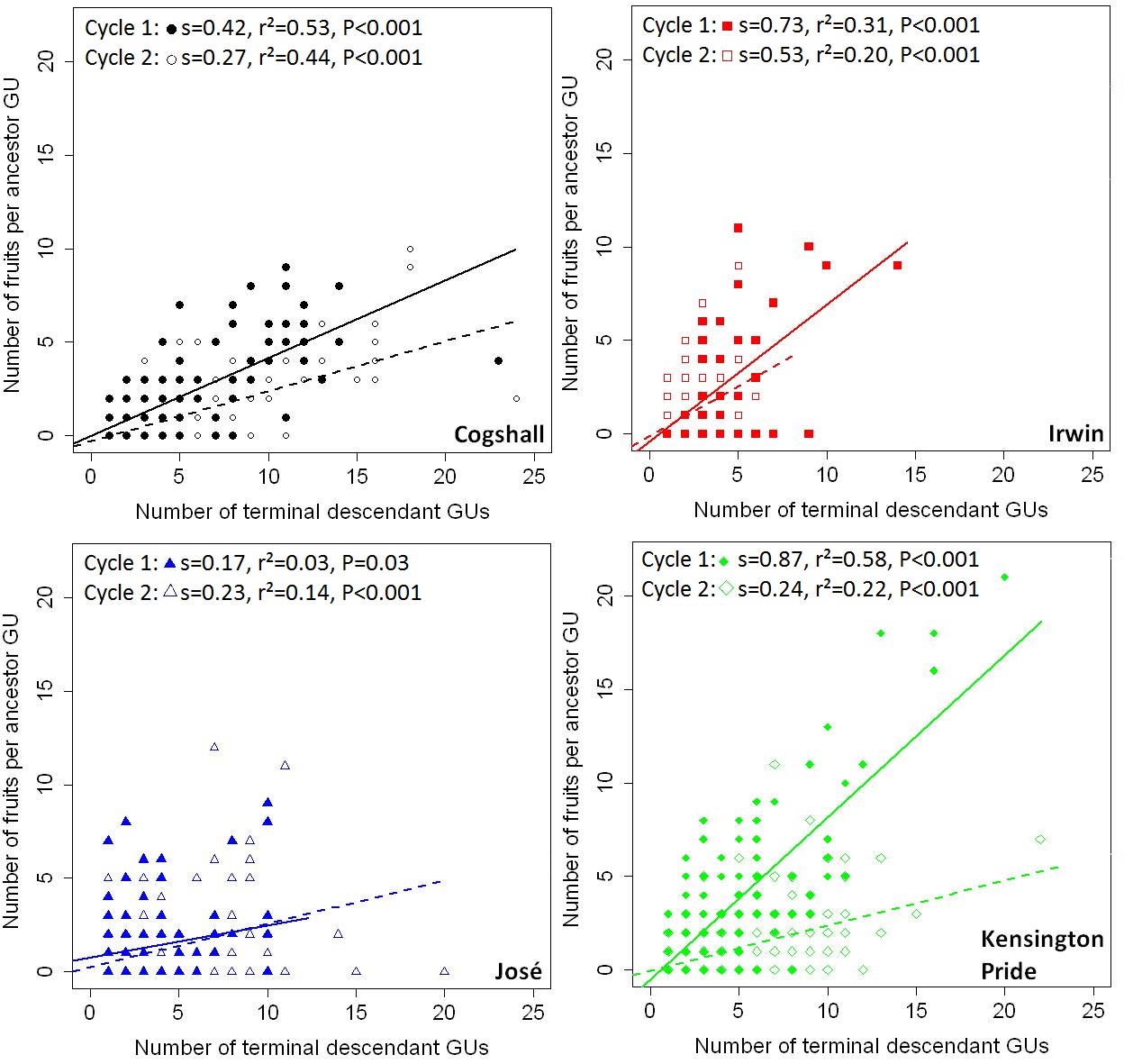

Supplement: FIGURE S3 — Relationships between the number of terminal descendant GUs produced by each ancestor GU and the number of fruits produced by these terminal descendant GUs for four mango cultivars, Cogshall, Irwin, José, and Kensington Pride, and two growing cycles. The slope (s), coefficient of determination (r2) and P-value associated with linear adjustments (cycle 1: solid line; cycle 2: dotted line) are given for each cycle. [file Image_3.JPEG]

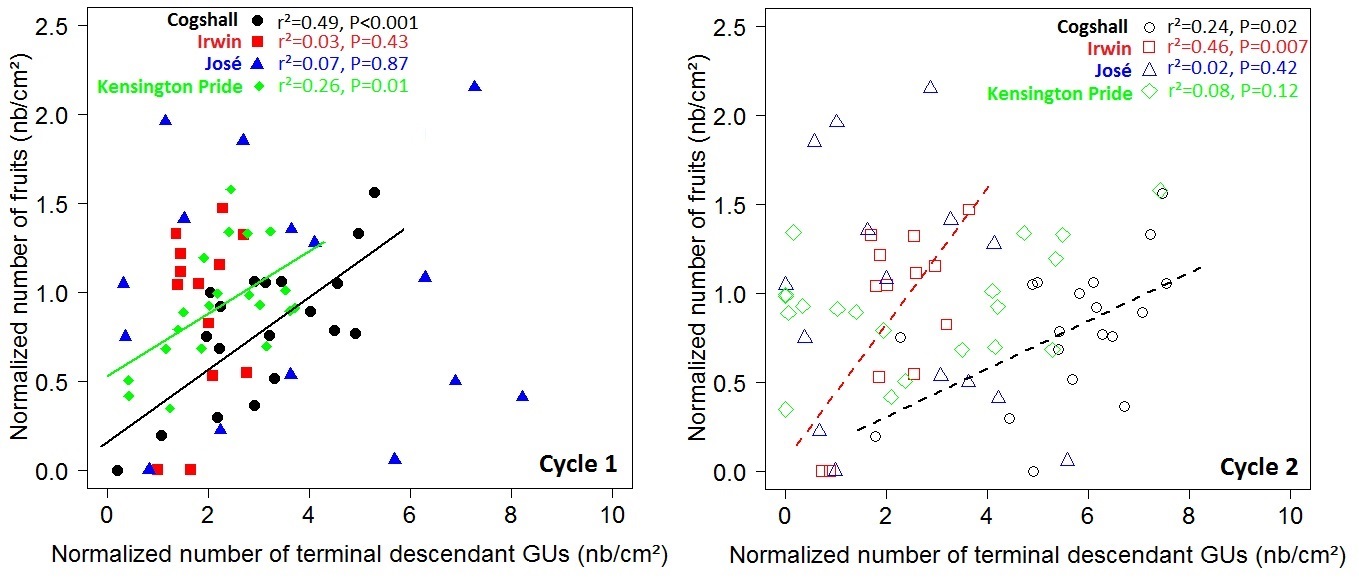

Supplement: FIGURE S4 — Relationships between the number of terminal descendant GUs produced during a cycle and the number of fruits produced during this cycle at the scaffold branch scale for four mango cultivars, Cogshall, Irwin, José, and Kensington Pride, and two growing cycles. The coefficient of determination (r2) and P-value associated with the linear adjustments are given in the figure. Regression lines are presented for significant relationships (P < 0.05). [file Image_4.jpg]
